# A Web-Based, Computer-Tailored Intervention to Reduce Alcohol Consumption and Binge Drinking Among Spanish Adolescents: Cluster Randomized Controlled Trial

**DOI:** 10.2196/15438

**Published:** 2020-01-24

**Authors:** José Manuel Martinez-Montilla, Liesbeth Mercken, Hein de Vries, Math Candel, Joaquín Salvador Lima-Rodríguez, Marta Lima-Serrano

**Affiliations:** 1 Department of Nursing School of Nursing, Physiotherapy and Podiatry University of Seville Seville Spain; 2 Department of Health Promotion Maastricht University Maastricht Netherlands; 3 Care and Public Health Research Institute Maastricht Netherlands; 4 Department of Methodology and Statistics Maastricht University Maastricht Netherlands

**Keywords:** adolescents, alcohol consumption, binge drinking, cluster randomized controlled trial, computer tailoring

## Abstract

**Background:**

Alcohol consumption, including binge drinking (BD) and heavy episodic drinking (HED), is one of the leading risk factors among Spanish adolescents leading to significant social, health, and economic consequences. Reduction of BD and HED in adolescents can be achieved using Web-based, computer-tailored (CT) interventions, providing highly personalized feedback that is adapted to a person’s individual characteristics and needs. Randomized controlled trials assessing the effects of tailored BD reduction programs among Spanish adolescents are scarce.

**Objective:**

The aim of this study was to test the effectiveness of the Web-based, CT intervention Alerta Alcohol, aimed at the prevention of BD in Spanish adolescents. As a secondary outcome, effects on HED, weekly consumption, and any consumption were also assessed. The adherence and process evaluation were assessed.

**Methods:**

A cluster randomized controlled trial conducted among 15 Spanish schools was developed. Each school was randomized into either an experimental condition (EC) (N=742) or a control condition (CC) (N=505). Finally, 351 participants for the EC and 261 for the CC were included in the analysis (N=612). Baseline assessment took place in January and February 2017. Demographic variables and alcohol use were assessed at baseline. Follow-up assessment of alcohol use took place 4 months later in May and June 2017. Participants were compared according to their randomization group (EC versus CC). After the baseline assessment, participants in the EC started the intervention, which consisted of short stories about BD, in which CT feedback was based on the I-Change Model for behavior change. Participants in the CC group only received the baseline questionnaire. Effects of the intervention were assessed using a three-level mixed logistic regression analysis for BD, HED, and any consumption, and a three-level mixed linear regression analysis for weekly consumption.

**Results:**

In total, 1247 adolescents participated in the baseline assessment and 612 participated in the follow-up assessment; the attrition rate was 50.92%. The intervention was effective in reducing HED among adolescents; the odds of HED in the CC was nine times that in the experimental condition (*P*=.04). No effects were found for BD, weekly consumption, and any consumption. Process evaluations revealed that the adolescents were satisfied with the program (68.8%), would use the program again (52.9%), and would recommend it to someone else (62.8%). Females and non-binge drinkers showed better responses in the process evaluation.

**Conclusions:**

Our intervention was effective regarding HED but not regarding BD, weekly consumption, and any consumption. It may be that limiting alcohol consumption to prevent HED was easier in the Spanish context than it was to carry out further steps, such as reducing other patterns of alcohol consumption. Hence, additional actions are needed to accomplish these latter goals, including community approaches and policy actions aimed at denormalizing alcohol consumption among Spanish adolescents.

**Trial Registration:**

ClinicalTrials.gov NCT03288896; https://clinicaltrials.gov/ct2/show/NCT03288896

**International Registered Report Identifier (IRRID):**

RR2-10.1186/s12889-018-5346-4

## Introduction

Alcohol consumption is one of the leading risk factors for mortality and disease worldwide [1,2], with significant social, health, and economic consequences [3], and is the leading risk factor globally for both death and disability-adjusted life years [4]. In addition, the most common risky drinking behavior among adolescents is binge drinking (BD), which consists of alcohol consumption that results in a blood alcohol concentration of 0.08 g/dL or more, within a period 2 hours [5,6]. In men, blood alcohol concentrations of more than 0.08 g/dL typically occur after consuming five or more standard drink units (SDUs), or standard glasses of alcohol, in about 2 hours; in women, this occurs after consuming four or more SDUs in about 2 hours [5,7]. In other words, BD occurs with the consumption of four or more and five or more SDUs of alcohol by women and men, respectively, in a short space of time or during a single occasion [5-8]. The lack of consensus as to what is considered an SDU could be the result of cross-country variability of criteria regarding the amount of alcohol consumption per episode [6,9,10]. However, the above definition, which is used in our study, is consistent with the Spanish epidemiological data from the survey Encuesta Sobre Uso de Drogas en Estudiantes de Enseñanzas Secundarias (ESTUDES) [8]. The fact is that BD is associated with detrimental long- and short-term consequences, since it affects neurocognitive development and leads to physical, psychological, and social alterations. In addition, BD has been associated with traffic accidents, violence, homicide, suicide, early sexual contact, school failure, mental illnesses, and delinquency, among other issues [3,11-15].

Moreover, heavy episodic drinking (HED) has been defined as the consumption of 10 or more glasses of alcohol on at least one occasion in the previous week [16,17]. This pattern of alcohol consumption is believed to be a serious problem in Western society, with major psychological, social, and economic consequences [17,18]. Similarly, HED has been linked to several problematic behaviors, such as an increased risk of unplanned sexual activity; increased risk of injury [19]; being more likely to engage in delinquent acts, including fighting, truancy from school, stealing, or driving while intoxicated[17]; as well as later alcohol abuse and dependence or illegal drug use [20].

The 2018 national Spanish survey ESTUDES showed that 75.6% of adolescents between 14 and 18 years of age drank alcohol in the last 12 months [8], a slightly lower number compared to that reported in the 2015 European School Survey Project on Alcohol and Other Drugs (ESPAD) [21]. Furthermore, in Europe, 35% of 15-16-year-old adolescents reported BD [21], while in Spain, the percentage stood at 32.2% of students between 14 and 18 years of age [8]. The national Spanish survey showed that the prevalence of BD at the age of 16 was 37.0%, which was twice that of 14-year-olds (13.2%), and BD reached 47.5% at the age of 17 [8]. Not one of the previous reports shows data on HED; however, Best et al [17] found a prevalence of 32% of HED among 14-16-year-old adolescents in the United Kingdom. These figures highlight the importance of preventing these different patterns of alcohol consumption in adolescents.

In the prevention area, computer- and Internet-based interventions are increasingly used as platforms for health promotion, including interventions aimed at reducing alcohol consumption [16,22-26]. Thus, the reduction of alcohol use and BD in adolescents could be achieved with the help of Web-based, computer-tailored (CT) interventions [16,27]; these could provide highly personalized feedback to individuals whose behaviors and opinions would be previously assessed on the basis of their answers to questionnaires, using data-driven decision rules that produce personalized feedback automatically from a database [28]. Some studies have shown that tailored advice helps to effectively change health behaviors and their determinants [16,23,24,27,29-31], even showing it to be cost-effective [32], although their effect sizes were generally small to medium [16,33]. However, Web-based, CT interventions usually have low adherence and high attrition rates [16,34,35], including over 50% [16,27,34,36], causing significant negative consequences, such as a reduced ability to reveal intervention effects [37].

The *Alerta Alcohol* program is the first Spanish program to be implemented that consists of a dynamic, Web-based, CT intervention in a school environment aimed at the prevention of alcohol consumption and BD in Spanish adolescents. This is a cultural adaptation of the Dutch program carried out by Jander et al [16,38]; however, in the Spanish context, no similar study has targeted these issues to date, and using CT technology at the high school level is still very rare [39]. Moreover, Alerta Alcohol tries to improve program adherence and minimize dropout in an attempt to overcome the limitations of previous studies. This program uses different strategies to accomplish this, such as developing a dynamic intervention with stories adapted to gender and age [23,39,40], based on the feedback from focus groups with adolescents (paper not yet published), or by carrying out the majority of the study at schools as part of the health promotion curriculum.

The aim of this study was to test the effectiveness of the Web-based, CT intervention Alerta Alcohol. We assessed the effects of the intervention on *BD*, *HED*, *weekly consumption*, and *any consumption*.

## Methods

### Ethics Committee Approval

The intervention was carried out according to bioethical guidelines; the students needed to answer the questionnaires themselves and confidentiality was guaranteed. Active informed consent was used. The project was approved the Bioethical Committee of Andalusia, Spain, and was registered on August 4, 2015 (registration number: PI-0031-2014). In addition, this trial was retrospectively registered at ClinicalTrials.gov on September 19, 2017 (NCT03288896). The intervention was not registered prospectively because our organization did not require it. However, the intervention was not modified with respect to the study protocol.

### Study Design

We conducted a cluster randomized controlled trial, with one experimental condition (EC) and one waiting-list control condition (CC); high schools were randomized into these two groups. Participants completed a baseline (ie, pretest) evaluation and a final (ie, posttest) evaluation performed 4 months after the intervention.

Participants were compared based on their randomization groups: EC versus CC. The EC group received the online intervention that contained CT feedback. The CC group only filled in the online baseline questionnaire. Both groups were given an online follow-up assessment after 4 months; they completed the same questionnaire that was used in the baseline assessment. The study took place in Spain between January and June 2017. The Consolidated Standards of Reporting Trials (CONSORT) guidelines were followed [41] (see Multimedia Appendix 1).

### Participants and Procedure

Participants were randomly selected from the group of students belonging to the public school system; this group included students in their fourth year of compulsory secondary education (CSE), those in the first year of their baccalaureate program, and those in the first year of continuing education or vocational training (VT), which is equivalent to 10th, 11th, and 12th grades in the United States, respectively. The randomization process was undertaken by two researchers from the team (JMMM and MLS) using a computer software randomization device to avoid contamination. First, we randomly selected at least two schools from each of the eight provinces in Andalusia in Southern Spain. If schools agreed to participate, the inclusion criteria were checked. If they did not agree to participate, we randomized other schools in the same province until at least two schools in each province were included; in total, we contacted 37 Andalusian schools.

The schools were informed of the objective of the study and the sessions of the intervention. Participation by each school was confirmed by email, telephone, or, when necessary, by a visit. A formal letter and an information folder were sent to teachers and coordinators at each school, where they were provided with contact details and the study website address [42], as well as a manual with frequently asked questions that may occur during the program.

Finally, after 16 high schools—two from each province in Andalusia—accepted, they were randomly assigned to either the EC group or the CC group, taking care that the intervention groups (ie, EC groups) were matched with a province. Within each school, all classes that met the inclusion criteria were invited to participate in the study.

**Figure 1:**
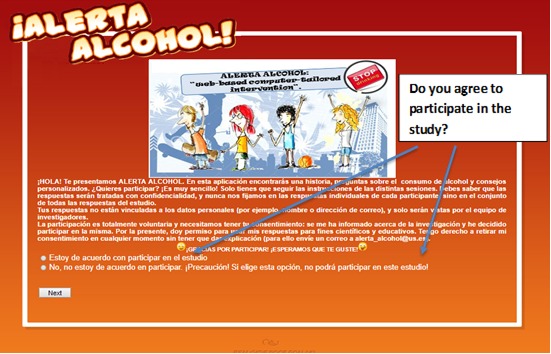
Screenshot of the informed consent page from the Alerta Alcohol website.

The CC schools were on a waiting list and received the intervention voluntarily once the study was completed. The selected schools were not blinded to their groups, since the EC group needed to schedule a total of four sessions during school hours. The adolescents were recruited from schools through their teachers and counselors. Adolescent participants and their parents had to sign and return the informed consent form to agree to take part in this scientific study. When starting the intervention, participants were asked to visit the study website and create an account. Within their account, they selected their school and were assigned to one of the conditions: CC or EC. Before starting with the baseline questionnaire, students gave informed consent by checking the acceptance box on the first page of the website. If he or she did not wish to participate, or refused to provide informed consent, he or she could select the option *I do not wish to participate in this study*. In this case, he or she was thanked and could leave the website. Participants could, however, also access the website at another time if they wanted to (see Figure 1). All students enrolled gave consent through active informed consent for the use of their data for scientific research and publication. Those who were underage were asked that their parents complete an informed consent form.

### Inclusion and Exclusion Criteria

The target group for the Alerta Alcohol program consisted of adolescents aged 15-19 years old that were enrolled in fourth-year CSE, first-year baccalaureate programs, or first-year VT. All students enrolled had Internet access at schools and in their homes. Those with language difficulties or those who had previously participated in prevention programs of BD were excluded. To check the inclusion criteria, a researcher was present at the pretest.

In addition, the inclusion criteria for schools were as follows: (1) public secondary schools from Andalusia, (2) schools belonging to provincial capitals, and (3) schools with access to the Internet and an equipped information and communication technology room available for students.

### Intervention

**Figure 2:**
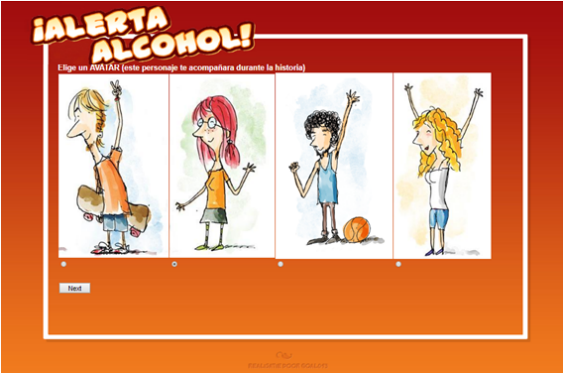
Screenshot of the page for choosing an avatar from the Alerta Alcohol website.

**Figure 3:**
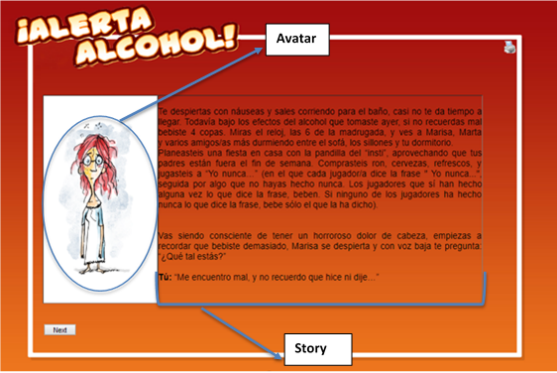
Screenshot of example stories for a girl from the Alerta Alcohol website.

Alerta Alcohol consists of short stories in which the main character binge drank the night before and his or her friends talk with him or her about what happened the night before. The drinking event took place in three scenarios: at home, at a celebration, and in a public place. The stories were designed based on the results of a focus group study (paper not yet published) and were adapted to the gender of the participant. Participants could choose an avatar and the names of the characters in the stories (see Figures 2 and 3).

First, the stories were presented and questions and tailored messages were shown, which were designed to reduce alcohol consumption and BD. Concise, direct, and personalized relevant messages were delivered to promote participation in the intervention [16,38,40]. The messages were customized with the names of the participants; elements such as repetition of the answers were used to show respect and empathy, counter persuasion, introduce social modelling and new beliefs, and reinforce positive behaviors and motivational feedback [28,43]. These messages were based on the I-Change Model, in which the central concepts are attitude, social influences, self-efficacy, and action planning [39,44]. In each scenario, self-efficacy is reinforced and action plans are offered to the adolescent in order to reject alcohol and BD in the specific scenario. In addition, we developed questions and tailored messages aiming to increase self-esteem and awareness of factors such as the acknowledgement of risk perception of alcohol consumption and BD.

Based on a previous focus group study, we concluded that it was necessary to favor premotivational and motivational components associated with BD; modification of beliefs; and risk perception and expectations, or those associated with the effects of alcohol experimentation; as well as to promote healthy self-esteem and self-efficacy. Gender differences were also taken into account (paper not yet published). In addition, a Delphi group study for the cultural adaptation study and the pilot study was developed, which allowed us to shorten and rewrite the feedback messages to make them more appealing to our target group (paper not yet published).

The intervention was carried out over six sessions. All students had access to a computer in their school’s computer room. After registering themselves online, all students had to fill out a baseline questionnaire (ie, pretest), which was supervised by a researcher. The following week, each student from the EC group continued with the intervention by logging in to the website; if needed, they were assisted by their teacher. The EC group members attended a second and third session held at school. There was a 1-2-week period between sessions; when possible, the schedule, which was planned with the researcher, was developed within the school curriculum (ie, class time). Students had two booster sessions at their homes 1 week after the third session. The fourth session was called *The Challenge*, where adolescents could accept the challenge of not drinking or at least not binge drink at an upcoming drinking event; the program reminded students again of the advice and action plans for this type of event. In the fifth session, 2 days after the drinking event, the program evaluated the challenge to determine whether or not the participants drank or at least whether they engaged in BD during this drinking event. At the end of the study, all students had to complete the sixth session at school (ie, the follow-up questionnaire), which was carried out throughout May and June 2017 (see Table 1). Each session took approximately 1 hour. The CC group only has two sessions: baseline data collection in January and February 2017 and the follow-up questionnaire (ie, posttest) 4 months later. A research technician was present for the pretest and posttest questionnaires at the schools to collaborate with the teachers and to optimize follow-up rates. Moreover, the researcher monitored the intervention by phone call or, if necessary, by visiting the schools in the EC group. Participation reminders were also sent via email when participants had not finished the intervention procedures, so they could complete them outside of school. Because of the nature of study, data were not anonymous. However, confidentiality was ensured through proper data management and security, according to the European General Data Protection Regulation (GDPR). This information was enclosed with the consent form. A detailed description of the development and content of the intervention is available in the protocol of this study [39].

**Table 1:** Alerta Alcohol program structure.

Location and session		Measures
**At school**		
	1	Initial questionnaire
	2	Scenario 1: at home Knowledge and risks Attitude: pros and cons Self-efficacy and action plans
	3	Scenario 2: celebrations Self-esteem Social modelling Self-efficacy and action plans
		Scenario 3: public places Social norms Social pressures Self-efficacy and action plans
**At student’s home**		
	4	Booster session: The Challenge
	5	Evaluation of The Challenge
**At school**		
	6	Final questionnaire

### Measures

#### Overview

Multiple guidelines for how drinking should be measured in surveys have been proposed; however, whether they are consistent in their recommendations has not been considered to date [45]. In this study, a Spanish validated version of the self-administered online questionnaire was used [46], which was adapted from a previous study carried out on Dutch adolescents [38]. A better description of the concepts and variables can be found in the study protocol [39] and in Multimedia Appendix 2.

#### Demographics

Social demographic variables were assessed at baseline, which consisted of gender (1=male and 2=female), age (in years), educational level (1=CSE, 2=baccalaureate, and 3=VT), religion (1=Catholic, 2=Protestant/Evangelical, 3=Muslim/Islamic, 4=other religion, and 5=no religion), and ethnicity (1=Spanish and 2=other).

In addition, we used the Family Affluence Scale (FAS) to measure social status. The FAS consists of four different questions: “Does your family have a car or a van?” “Do you have your own room at home?” “During the last 12 months, how many times have you gone on holiday with your family?” and “How many computers does your family have?” [47]. The FAS was transformed into three categories, where students could belong to the low level (0-2 points), middle level (3-5 points), or high level (6-9 points).

The Family Apgar Test was used to measure self-perception on familiar functional status (alpha=.778). It consists of five questions answered on a 3-point Likert scale (0=almost never, 1=sometimes, and 2=almost always); the resulting score was dichotomized into dysfunctional family status (score 0-6) and functional family status (score 7-10) [48].

#### Binge Drinking, Heavy Episodic Drinking, Weekly Consumption, and Any Consumption

BD was assessed using an open-ended question on how many BD occasions they participated in during the previous 30 days (eg, for girls, “How often did you drink 4 or more standard glasses of alcohol on one occasion in the previous 30 days?”; for boys, the number of drinks was 5 or more). A figure showing different standard drinks was shown to make the concept more comprehensible [16]. This variable was then dichotomized (0=reported no BD and 1=reported BD).

For HED, participants were dichotomized into two groups: those who consumed 10 or more glasses of alcohol on at least one occasion in the previous week (1=HED) and those who did not (0=no HED) [16,17].

For weekly consumption, we assessed how many glasses of alcohol students drank each day during the last week. Based on this information, we calculated the total number of glasses consumed in the past week [16].

Any consumption was calculated using the question “On which days of the past week did you drink alcohol?” Possible answers were as follows: “Monday to Sunday”; “I haven’t drunk in the past week”; and “I have never drunk alcohol.” This variable was dichotomized into two groups (0=no and 1=yes).

#### Process Evaluation and Adherence

To assess adherence, the number of intervention sessions attended by the participants at schools was registered. Furthermore, after completing each session, we asked respondents whether they have been reading the advice, whether the intervention was useful, whether the content was credible and appropriate, and whether the advice was interesting, understandable, too long or short, and personally relevant; responses were based on a 5-point Likert scale (eg, 1=totally disagree and 5=totally agree). We also assessed whether the advice increased their knowledge, changed their risk perception, changed their attitude, or improved their skills to prevent BD. For the analysis of these questions, answers were converted into three categories: 1=totally disagree/partially disagree, 2=neither agree nor disagree, and 3=totally agree/partially agree.

Finally, we also assessed general satisfaction with the program using a 5-point Likert scale. This question was converted into three categories: 1=very dissatisfied/dissatisfied, 2=neither satisfied nor dissatisfied, and 3=very satisfied/satisfied. Using a 5-point Likert scale, we also assessed whether, given the opportunity, they would use the program again and whether they would recommend the program to others (eg, 1=totally disagree and 5=totally agree) [49].

### Power Analyses

The primary outcome was the difference in binge-drinking occasions in the previous 30 days in the EC group compared with the CC group. According to the 2016 national Spanish survey ESTUDES [50], the prevalence of adolescent BD within a previous 30-day timeframe was 32.2%. It is estimated that the intervention reduces consumption to 22%. Requiring a statistical power of .80 for a two-sided test with a type I error rate of alpha=.05, 309 subjects were required for the CC group and 309 were required for the EC group (ie, 618 participants in total); G*Power, version 3.1.9.2 (Heinrich-Heine-Universität Düsseldorf), was used for the statistical power analyses [51]. Following the study by Jander et al [38], a dropout rate of about 50% was anticipated; therefore, 1236 total subjects needed to be recruited.

### Statistical Analyses

Descriptive analyses were performed to describe the characteristics of the participants at baseline. Differences between the conditions in the baseline sample, as well as between consumers and nonconsumers—BD, HED, weekly consumption, and any consumption—were assessed via a *t* test for continuous variables and a chi-square test for categorical variables. Also, when the dependent variable was not normally distributed, the Mann-Whitney *U* test was used.

Since pupils were nested within classes in the study, and classes were nested within schools, in order to examine predictors of dropout versus nondropout, a three-level mixed logistic regression analysis was conducted. To test the effectiveness of the program, we also performed three-level mixed logistic regression analyses for the outcomes *BD*, *any consumption*, and *HED*, and a three-level mixed linear regression for the outcome *weekly consumption*. When variances of the random intercept at the school level and class level turned out to be zero, a standard logistic or linear regression was carried out. This turned out to be the case for the binary outcomes *any consumption*, *BD*, and *HED*.

To evaluate the effect, the intention-to-treat principle was followed. The independent variable of interest was included in the EC versus CC, and covariates were the outcome at baseline as well as several sociodemographic variables: gender, age, educational level, religion, ethnicity, Apgar score, and affluence level. Also, the interaction effects between intervention condition and all sociodemographic variables were entered as covariates into the analyses. To build the refined model, we first examined whether the covariance model of random effects could be simplified. In the second phase, the variables with the least statistical significance, provided their significance was above .10 (for interaction terms) or .05 (for main effect terms), were eliminated one by one from the model. However, the variable of central interest—EC versus CC—always remained in the model. To quantify the predictive power of the logistic regression models, Nagelkerke’s R^2^ was reported. Due to the high attrition rate (>50%) in the majority of variables in the follow-up questionnaire, thus hampering the validity of multiple imputation techniques, we decided not to use multiple imputation and we performed the analysis with pairwise deletion [52,53].

To study predictors of adherence, we also analyzed the associations between potential participant characteristics (ie, gender, age, educational level, religion, ethnicity, Apgar score, family affluence, and alcohol use at baseline) on the one hand and participation in the intervention (ie, adherent or not) on the other.

Finally, for the process evaluation a descriptive analysis was performed using chi-square tests to examine differences between males and females and between binge drinkers and non-binge drinkers. We used SPSS Statistics for Windows, version 21.0 (IBM Corp), for these analyses. The level of significance used for the main effects was alpha=.05 and for the interaction effects was alpha=.10. Also, effect sizes (odds ratios [ORs]) and 95% CIs were calculated.

## Results

### Participation and Attrition Rate

After contacting several schools, 16 accepted our invitation. Reasons for nonacceptance were as follows: the program was too time-consuming, the schools were involved in another drug-related program, or there were logistical problems, such as not having a computer room. Other schools did not respond to our phone calls and emails.

**Figure 4:**
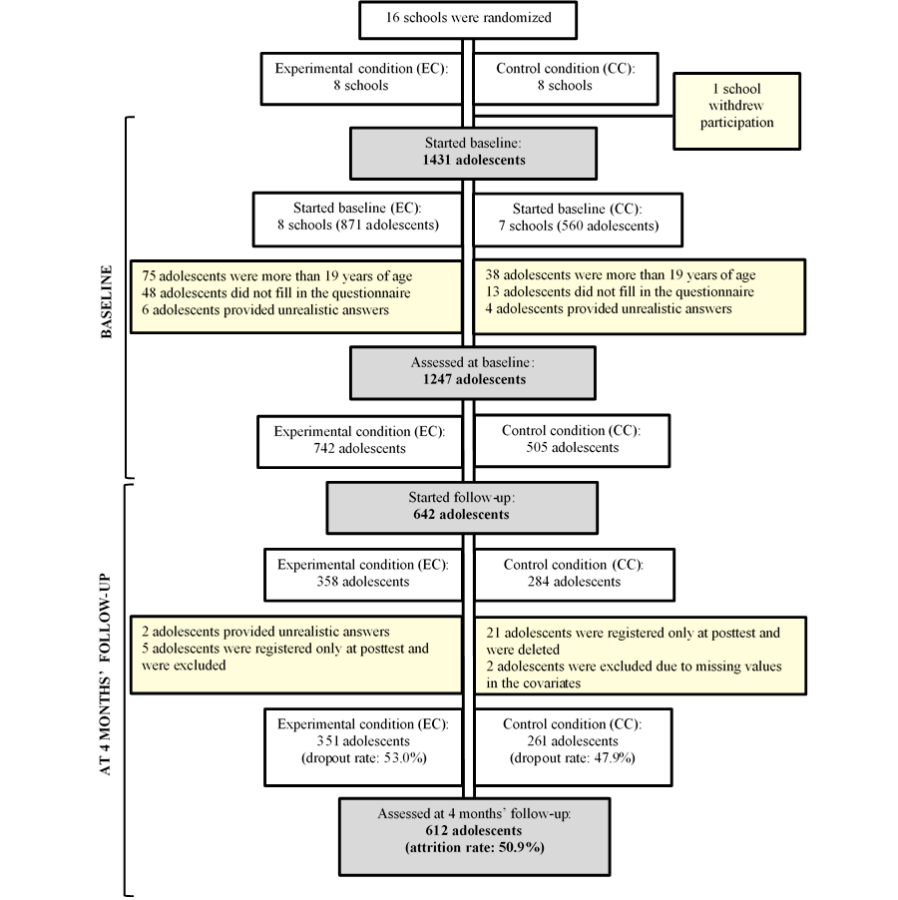
Flowchart of participant recruitment and dropouts in the Alerta Alcohol program.

In total, 16 schools were randomly assigned to either the EC or CC. However, one of the schools from the CC did not begin the baseline assessment and withdrew their participation due to logistical problems. In total, 1431 adolescents from 15 schools—8 EC and 7 CC—were requested to fill in the baseline questionnaire. Subsequently, 75 adolescents from the EC group and 38 from the CC group were removed because they were over 19 years of age, 48 students were removed from the EC group and 13 from the CC group because they did not fill in the questionnaire, and an additional 6 students from EC group and 4 from the CC group were removed because they provided unrealistic answers. In total, 1247 interviews were used at the baseline assessment—742 from the EC and 505 from the CC. Figure 4 shows the flowchart of participants at the participating schools.

All 15 schools participated in the completion of the 4-month follow-up questionnaire, with only 612 participants: 351 from the EC group and 261 from the CC group. There was a clear decrease in participation between the baseline assessment (N=742) and the follow-up assessment (N=351) in the EC group, resulting in an attrition rate of 50.92% (612/1247). Most schools had problems with their Wi-Fi or reported trouble finding a suitable date, due to final exams. Also, groups participating in VT were beginning their placements in companies around this time, so this was also a cause of dropout.

The dropout analyses showed that adolescents returning the follow-up questionnaires were significantly younger (*P*<.001) and had lower educational levels (*P*<.001). The adolescents that returned the questionnaires were less likely to be binge drinkers (*P*=.02) and had a lower weekly consumption (*P*=.01) (see Table 2). When a multilevel analysis was carried out, the older participants (OR 1.319, 95% CI 1.101-1.581, *P*=.003) and those who reported a higher weekly consumption (OR 1.045, 95% CI 1.004-1.087, *P*=.03) were more likely to drop out of the intervention.

**Table 2:** Differences in baseline characteristics between participants that returned and those that dropped out at the 4-month follow-up.

Variable (number of missing values)			Total (N=1247)		Returned (n=612)		Dropped out (n=635)		Test statistic		*P* value
Age (15-19-year-olds) (0), mean (SD)			16.32 (1.07)		16.11 (0.99)		16.53 (1.10)		*U*^a^=236280.5; *t*_1237.926_=–7.090		<.001; <.001
**Gender (0), n (%)**									**χ^2^_1_=0.5**		**.49**
	Male	586 (46.99)		282 (46.1)		304 (47.9)					
	Female	661 (53.01)		330 (53.9)		331 (52.1)					
**Educational level (0), n (%)**									**χ^2^_2_=58.8**		**<.001**
	Compulsory secondary education	545 (43.70)		313 (51.1)		232 (36.5)					
	Baccalaureate program	515 (41.30)		252 (41.2)		263 (41.4)					
	Vocational training	187 (15.00)		47 (7.7)		140 (22.1)					
**Religion (3), n (%)**									**χ^2^_4_=2.9**		**.57**
	Catholic	767 (61.65)		385 (62.9)		382 (60.4)					
	Protestant or Evangelical	26 (2.09)		11 (1.8)		15 (2.4)					
	Muslim or Islamic	18 (1.45)		9 (1.5)		9 (1.4)					
	Other	28 (2.25)		10 (1.6)		18 (2.8)					
	No religion	405 (32.56)		196 (32.1)		209 (32.9)					
**Ethnicity (3), n (%)**									**χ^2^_1_=1.2**		**.26**
	Spanish	1178 (94.69)		583 (95.4)		595 (94.0)					
	Non-Spanish	66 (5.31)		28 (4.6)		38 (6.0)					
**Apgar test (55), n (%)**									**χ^2^_1_=1.2**		**.28**
	Functional family	886 (74.33)		446 (75.7)		440 (73.0)					
	Dysfunctional family	306 (25.67)		143 (24.3)		163 (27.0)					
**Family affluence (49), n (%)**									**χ^2^_2_=0.9**		**.63**
	Low	33 (2.76)		16 (2.7)		17 (2.8)					
	Medium	387 (32.30)		183 (31.0)		204 (33.5)					
	High	778 (64.94)		391 (66.3)		387 (63.7)					
**Alcohol use (0)**											
	Binge drinking, n (%)	487 (39.05)		219 (35.8)		268 (42.2)		χ^2^_1_=5.6		.02	
	Heavy episodic drinking, n (%)	16 (1.28)		8 (1.3)		8 (1.3)		χ^2^_1_=0.005		.95	
	Weekly consumption, mean (SD)	1.45 (4.30)		1.22 (3.33)		1.68 (5.06)		*U*=206367; *t*_1098.665_=–1.929		.01; .054	
	Any consumption, n (%)	832 (66.72)		404 (66.0)		428 (67.4)		χ^2^_1_=0.4		.55	

^a^Mann-Whitney *U* test.

### Sample Characteristics

Baseline characteristics are shown in Table 3. We found that the adolescents in the EC group were older than those in the CC group (*P*<.001). We also found that the educational level of adolescents was higher in the CC group compared to the EC group (*P*=.002).

**Table 3:** Baseline characteristics of participants by condition.

Variable (number of missing values)		Total (N=1247)		Experimental group (n=742)		Control group (n=505)		Test statistic		*P* value	
Schools, n		15		8		7		N/A^a^		N/A	
Classrooms per school, n		62		38		24		N/A		N/A	
Students per classroom, mean (SD), range		N/A		19.53 (6.90), 25		21.04 (5.87), 26		N/A		N/A	
Age (15-19-year-olds) (0), mean (SD)		16.32 (1.07)		16.40 (1.06)		16.21 (1.06)		*U*^b^=208553; *t*_1245_=–3.150		<.001; .002	
**Gender (0), n (%)**								**χ^2^_1_=0.2**		**.67**	
	Male		586 (46.99)		345 (46.5)		241 (47.7)				
	Female		661 (53.01)		397 (53.5)		264 (52.3)				
**Educational level (0), n (%)**								**χ^2^_2_=12.1**		**.002**	
	Compulsory secondary education		545 (43.70)		321 (43.3)		224 (44.4)				
	Baccalaureate program		515 (41.30)		289 (38.9)		226 (44.8)				
	Vocational training		187 (15.00)		132 (17.8)		55 (10.9)				
**Religion (3), n (%)**								**χ^2^_4_=3.1**		**.55**	
	Catholic		767 (61.65)		462 (62.3)		305 (60.6)				
	Protestant or Evangelical		26 (2.09)		19 (2.6)		7 (1.4)				
	Muslim or Islamic		18 (1.45)		10 (1.4)		8 (1.6)				
	Other		28 (2.25)		15 (2.0)		13 (2.6)				
	No religion		405 (32.56)		235 (31.7)		170 (33.8)				
**Ethnicity (3), n (%)**								**χ^2^_1_=1.9**		**.17**	
	Spanish		1178 (94.69)		707 (95.4)		471 (93.6)				
	Non-Spanish		66 (5.31)		34 (4.6)		32 (6.4)				
**Apgar test (55), n (%)**								**χ^2^_1_=2.2**		**.14**	
	Functional family		886 (74.33)		516 (72.8)		370 (76.6)				
	Dysfunctional family		306 (25.67)		193 (27.2)		113 (23.4)				
**Family affluence (49)**								**χ^2^_2_=3.5**		**.17**	
	Low		33 (2.76)		17 (2.4)		16 (3.3)				
	Medium		387 (32.30)		244 (34.2)		143 (29.5)				
	High		778 (64.94)		452 (63.4)		326 (67.2)				
**Alcohol use (0)**											
	Binge drinking, n (%)		487 (39.10)		298 (40.2)		189 (37.4)		**χ^2^_1_=0.9**		**.33**
	Heavy episodic drinking, n (%)		16 (1.30)		8 (1.1)		8 (1.6)		χ^2^_1_=0.6		.44
	Weekly consumption, mean (SD)		1.45 (4.30)		1.47 (3.78)		1.42 (4.96)		*U*=190393.5 *t*_1245_=-0.2		.52 .84
	Any consumption, n (%)		832 (66.70)		486 (65.5)		346 (68.5)		χ^2^_1_=1.2		.27

^a^Not applicable.

^b^Mann-Whitney *U* test.

### Binge Drinking

At baseline, 40.2% (298/742) of adolescents in the EC group and 37.4% (189/505) in the CC group reported BD in the previous 30 days. At the follow-up session, 32.2% (113/351) of adolescents in the EC group and 33.0% (86/261) in the CC group reported BD. There was a reduction in BD in both conditions, but this reduction was only significant in the EC group: BD was reduced by 8.0% (OR 0.716, 95% CI 0.547-0.937, *P*=.02) in the EC group versus a reduction of 4.4% (OR 0.821, 95% CI 0.600-1.126, *P*=.22) in the CC group. However, in the logistic regression analysis, the intervention did not show a significant effect; although, in the CC group, the odds of BD were 1.1 times the odds of BD in the EC group. In addition, the analysis revealed that family affluence was marginally significant (*P*=.08); people who had medium family affluence (OR 3.365, 95% CI 1.058-10.704, *P*=.04) had a higher risk of BD than people who had high family affluence (see Tables 4 and 5).

**Table 4:** Effects of the Alerta Alcohol program on binge drinking, heavy episodic drinking, weekly consumption, and any consumption in the complete model.

Variable		Binge drinking^a^ (N=586)			Heavy episodic drinking^a^ (N=612)			Weekly consumption^b^ (N=589)				Any consumption^a^ (N=589)	
		OR^c^ (95% CI)	*P* value	OR (95% CI)		*P* value	B (SE)		*P* value	95% CI	OR (95% CI)		*P* value
Condition (control vs experimental)		1.106 (0.730-1.674)	.63	9.129 (1.107-75.259)		.04	–0.244 (0.385)		.53	–1.000-0.511	0.866 (0.506-1.484)		.60
Gender (male vs female)		N/A^d^	N/A	3.394 (0.669-17.223)		.14	N/A		N/A	N/A	N/A		N/A
**Family affluence**													
	Low versus high	3.365 (1.058-10.704)	.04	N/A		N/A	–1.503 (0.921)		.10	–3.313-0.306	3.401 (0.710-16.129)		.13
	Medium versus high	1.309 (0.841-2.036)	.23	N/A		N/A	0.410 (0.325)		.21	–0.228-1.048	1.801 (0.987-3.289)		.06
Consumption behavior pretest^e^		11.986 (7.951-18.069)	<.001	8.360 (0.820-85.239)		.07	0.502 (0.044)		<.001	0.416-0.589	59.518 (34.789-101.823)		<.001

^a^Logistic regression.

^b^Linear mixed regression.

^c^OR: odds ratio.

^d^Not applicable.

^e^Consumption behavior—binge drinking, heavy episodic drinking, weekly consumption, and any consumption—at the pretest evaluation.

**Table 5:** Fixed effects of the Alerta Alcohol program on binge drinking, heavy episodic drinking, weekly consumption, and any consumption in the complete model.

Variable		Binge drinking^a^ (N=586)		Heavy episodic drinking^a^ (N=612)			Weekly consumption^b^ (N=589)		Any consumption^a^ (N=589)		
		Statistic	*P* value		Statistic	*P* value	Statistic	*P* value		Statistic	*P* value
Condition		W^c^=0.225	.64		W=4.222	.04	F^d^=0.403	.53		W=0.271	.60
Family affluence		W=5.108	.08		N/A^e^	N/A	F=2.405	.09		W=5.443	.07
		*R*^2f^=.349	N/A		*R*^2^=.150	N/A	N/A	N/A		*R*^2^=.638	N/A
**Intracluster correlation (ICC)**											
	School level	N/A	N/A		N/A	N/A	ICC=0	N/A		N/A	N/A
	Class level	N/A	N/A		N/A	N/A	ICC=.046	N/A		N/A	N/A

^a^Logistic regression.

^b^Linear mixed regression.

^c^Wald test.

^d^F test.

^e^Not applicable.

^f^Nagelkerke’s *R*^2^.

### Heavy Episodic Drinking

At baseline, 1.1% (8/742) of adolescents in the EC group and 1.6% (8/505) in the CC group reported heavy episodic drinking in the previous 30 days. At the follow-up stage, 0.3% (1/351) of adolescents in the EC group and 2.7% (7/261) in the CC group reported heavy episodic drinking in the previous 30 days. The logistic regression analysis showed that the odds of heavy episodic drinking in the CC group was nine times the odds of heavy episodic drinking in the EC group (OR 9.129, 95% CI 1.107-75.259, *P*=.04).

### Weekly Consumption

At baseline, adolescents in the EC group and in the CC group drank a mean of 1.47 (SD 3.78) and 1.42 (SD 4.96) standard glasses of alcohol, respectively, in the previous week. At the follow-up stage, the mean was 1.64 (SD 3.66) and 1.39 (SD 4.12) standard glasses of alcohol in the EC group and in the CC group, respectively. The mixed linear regression analysis did not show any significant effects of the intervention.

### Any Consumption

At baseline, 65.5% (486/742) of adolescents in the EC group and 68.5% (346/505) in the CC group reported that they had never drunk alcohol. At the follow-up stage, 68.9% (242/351) of adolescents in the EC group and 65.5% (171/261) in the CC group reported that they had never drunk alcohol. In the logistic regression analysis, the intervention had no effect. Again, family affluence was marginally significant (*P*=.07), where participants with low family affluence (OR 1.801, 95% CI 3.289-0.987, *P*=.06) had a higher probability of drinking alcohol than those with high family affluence.

### Adherence

After the baseline (ie, first) session, of the 742 adolescents who were randomized into the EC, only 461 (62.1%) started the second session at school and only 350 (47.2%) returned for the third session at school. Only 23 adolescents out of 742 (3.1%) returned for the fourth session at home and 8 (1.1%) returned for the fifth session at home.

We assessed the predictors of adherence, which were as follows: educational level (*P*=.009) (with students enrolled in VT showing less adherence than baccalaureate program students: beta=–0.882, *P*=.002), ethnicity (Spanish versus other: beta=1.142, *P*=.04), and not engaging in BD in the previous 30 days at baseline (beta=–0.546, *P*=.03) (see Table 6).

**Table 6:** Predictors of adherence where both sessions were completed.

Variable^a^		Βeta	SE	Exp(beta)	*P* value
Gender (female)		0.319	0.229	1.376	.16
Age		–0.140	0.138	0.870	.31
**Educational level (baccalaureate)**					**.009**
	Vocational training	–0.882	0.287	0.414	.002
	Compulsory secondary education	–0.201	0.340	0.818	.55
**Religion (no religion)^b^**					**.30**
	Others	0.005	0.256	1.005	.98
	Catholic	0.950	0.627	2.586	.13
Ethnicity (others)		1.142	0.566	3.132	.04
**Apgar test (family dysfunction)**		0.162	0.287	1.176	**.57**
	High family affluence				.19
	Medium family affluence	1.313	1.091	3.717	.23
	Low family affluence	0.385	0.257	1.470	.13
Binge drinking		–0.546	0.250	0.579	.03

^a^Reference category of categorical variables is indicated between brackets.

^b^Religion was entered as three categories: no religion, others, and Catholic.

### Process Evaluation

Of the EC group members, 295 participants returned the questionnaire. In total, 50.8% (150/295) of students reported that the sessions were too long. Even though 76.1% (223/293) of students said the advice content was credible, 72.0% (211/293) stated that it was understandable, 63.1% (185/293) stated that it was useful, and 60.0% (177/295) stated that the advice was interesting. Furthermore, 68.8% (203/295) of students were satisfied with the program, 52.9% (155/293) would use the program again, and 62.8% (184/293) recommended the program to someone else (see Tables 7 and 8).

**Table 7:** Process evaluation of the Alerta Alcohol program: responses from participants in the intervention group by gender.

Variable (number of missing values)		Total (N=351), n (%)	Male (N=149), n (%)	Female (N=202), n (%)	χ^2^_2_	*P* value
**Overall satisfaction (56)**					**1.0**	**.61**
	Very dissatisfied/dissatisfied	35 (11.9)	18 (14.0)	17 (10.2)		
	Neither satisfied nor dissatisfied	57 (19.3)	25 (19.4)	32 (19.3)		
	Very satisfied/satisfied	203 (68.8)	86 (66.7)	117 (70.5)		
**Length of session: too long (56)**					**2.4**	**.31**
	Totally disagree/partially disagree	49 (16.6)	19 (14.7)	30 (18.1)		
	Neither agree nor disagree	96 (32.5)	48 (37.2)	48 (28.9)		
	Totally agree/partially agree	150 (50.8)	62 (48.1)	88 (53)		
**Length of session: too short (56)**					**1.0**	**.60**
	Totally disagree/partially disagree	88 (29.8)	38 (29.5)	50 (30.1)		
	Neither agree nor disagree	101 (34.2)	48 (37.2)	53 (31.9)		
	Totally agree/partially agree	106 (35.9)	43 (33.3)	63 (38.0)		
**Interest in advice (56)**					**1.0**	**.60**
	Totally disagree/partially disagree	52 (17.6)	26 (20.2)	26 (15.7)		
	Neither agree nor disagree	66 (22.4)	28 (21.7)	38 (22.9)		
	Totally agree/partially agree	177 (60.0)	75 (58.1)	102 (61.4)		
**Content credibility (58)**					**4.5**	**.10**
	Totally disagree/partially disagree	24 (8.2)	15 (11.7)	9 (5.5)		
	Neither agree nor disagree	46 (15.7)	22 (17.2)	24 (14.5)		
	Totally agree/partially agree	223 (76.1)	91 (71.1)	132 (80.0)		
**Advice was useful (58)**					**6.9**	**.03**
	Totally disagree/partially disagree	44 (15.0)	27 (21.1)	17 (10.3)		
	Neither agree nor disagree	64 (21.8)	28 (21.9)	36 (21.8)		
	Totally agree/partially agree	185 (63.1)	73 (57.0)	112 (67.9)		
**Advice was understandable (58)**					**>9.1**	**.01**
	Totally disagree/partially disagree	25 (8.5)	18 (14.1)	7 (4.2)		
	Neither agree nor disagree	57 (19.5)	25 (19.5)	32 (19.4)		
	Totally agree/partially agree	211 (72.0)	85 (66.4)	126 (76.4)		
**Advice was appropriate (58)**					**4.3**	**.12**
	Totally disagree/partially disagree	49 (16.7)	28 (21.9)	21 (12.7)		
	Neither agree nor disagree	73 (24.9)	30 (23.4)	43 (26.1)		
	Totally agree/partially agree	171 (58.4)	70 (54.7)	101 (61.2)		
**Improved knowledge (58)**					**9.5**	**.009**
	Totally disagree/partially disagree	46 (15.7)	29 (22.7)	17 (10.3)		
	Neither agree nor disagree	72 (24.6)	25 (19.5)	47 (28.5)		
	Totally agree/partially agree	175 (59.7)	74 (57.8)	101 (61.2)		
**Changed attitude (58)**					**4.1**	**.13**
	Totally disagree/partially disagree	54 (18.4)	30 (23.4)	24 (14.5)		
	Neither agree nor disagree	90 (30.7)	39 (30.5)	51 (30.9)		
	Totally agree/partially agree	149 (50.9)	59 (46.1)	90 (54.5)		
**Changed risk perception (58)**					**5.7**	**.06**
	Totally disagree/partially disagree	49 (16.7)	29 (22.7)	20 (12.1)		
	Neither agree nor disagree	81 (27.6)	33 (25.8)	48 (29.1)		
	Totally agree/partially agree	163 (55.6)	66 (51.6)	97 (58.8)		
**Improved skills to reduce binge drinking (58)**					**7.6**	**.02**
	Totally disagree/partially disagree	45 (15.4)	28 (21.9)	17 (10.3)		
	Neither agree nor disagree	86 (29.4)	33 (25.8)	53 (32.1)		
	Totally agree/partially agree	162 (55.3)	67 (52.3)	95 (57.6)		
**Would use the program again (58)**					**5.2**	**.07**
	Totally disagree/partially disagree	49 (16.7)	28 (21.9)	21 (12.7)		
	Neither agree nor disagree	89 (30.4)	33 (25.8)	56 (33.9)		
	Totally agree/partially agree	155 (52.9)	67 (52.3)	88 (53.3)		
**Would recommend the program to someone else (58)**					**6.1**	**.047**
	Totally disagree/partially disagree	37 (12.6)	22 (17.2)	15 (9.1)		
	Neither agree nor disagree	72 (24.6)	25 (19.5)	47 (28.5)		
	Totally agree/partially agree	184 (62.8)	81 (63.3)	103 (62.4)		

Regarding gender, more females, compared to males, reported that the advice was useful (*d*=0.294, 95% CI 0.062-0.527, *P*=.03), understandable (*d*=0.316, 95% CI 0.083-0.548, *P*=.01), improved their knowledge (*d*=0.212, 95% CI 0.019-0.443, *P*=.009), changed their risk perception (*d*=0.236, 95% CI 0.005-0.468, *P*=.06), and improved their skills (*d*=0.229, 95% CI 0.003-0.460, *P*=.02). In general, more females, compared to males, more frequently reported that they would recommend the program to someone else (*d*=0.102, 95% CI 0.129-0.333, *P*=.047); this was a mixed result, since males also more often answered *totally agree/partially agree* for this question, as well as there being more males who answered *totally disagree/partially disagree*. Therefore, we could say that males are somewhat more extreme regarding their opinion on this question.

Regarding BD, more non-binge drinkers reported the advice to be credible (*d*=0.199, 95% CI 0.039-0.437, *P*=.03). On the other hand, more binge drinkers reported the advice to be useful (*d*=0.040, 95% CI 0.197-0.277, *P*=.02), to be appropriate for them (*d*=0.293, 95% CI 0.054-0.531, *P*=.01), and to improve their knowledge (*d*=0.068, 95% CI 0.170-0.305, *P*=.02), as well as that the advice changed their risk perception (*d*=0.078, 95% CI 0.159-0.315, *P*=.02).

**Table 8:** Process evaluation of the Alerta Alcohol program: responses from participants in the intervention group by binge-drinking status.

Variable (number of missing values)		No binge drinking (N=198), n (%)		Binge drinking (N=153), n (%)		χ^2^_2_		*P* value	
**Overall satisfaction (56)**						**0.7**		**.69**	
	Very dissatisfied/dissatisfied		24 (12.9)		11 (10.1)				
	Neither satisfied nor dissatisfied		34 (1.3)		23 (21.1)				
	Very satisfied/satisfied		128 (68.8)		75 (68.8)				
**Length of session: too long (56)**						**5.9**		**.053**	
	Totally disagree/partially disagree		37 (19.9)		12 (11.0)				
	Neither agree nor disagree		53 (28.5)		43 (39.4)				
	Totally agree/partially agree		96 (54.6)		54 (49.5)				
**Length of session: too short (56)**						**6.0**		**.049**	
	Totally disagree/partially disagree		63 (33.9)		25 (22.9)				
	Neither agree nor disagree		55 (29.6)		46 (42.2)				
	Totally agree/partially agree		68 (36.6)		38 (34.9)				
**Interest in advice (56)**						**1.7**		**.43**	
	Totally disagree/partially disagree		36 (19.4)		16 (14.7)				
	Neither agree nor disagree		38 (20.4)		28 (25.7)				
	Totally agree/partially agree		112 (60.2)		65 (59.6)				
**Content credibility (58)**						**7.3**		**.03**	
	Totally disagree/partially disagree		15 (8.1)		9 (8.3)				
	Neither agree nor disagree		21 (11.4)		25 (23.1)				
	Totally agree/partially agree		149 (80.5)		74 (68.5)				
**Advice was useful (58)**						**7.6**		**.02**	
	Totally disagree/partially disagree		33 (17.8)		11 (10.2)				
	Neither agree nor disagree		32 (17.3)		32 (29.6)				
	Totally agree/partially agree		120 (64.9)		65 (60.2)				
**Advice was understandable (58)**						**2.4**		**.30**	
	Totally disagree/partially disagree		17 (9.2)		8 (7.4)				
	Neither agree nor disagree		31 (16.8)		26 (24.1)				
	Totally agree/partially agree		137 (74.1)		74 (68.5)				
**Advice was appropriate (58)**						**8.7**		**.01**	
	Totally disagree/partially disagree		40 (21.6)		9 (8.3)				
	Neither agree nor disagree		43 (23.2)		30 (27.8)				
	Totally agree/partially agree		102 (55.1)		69 (63.9)				
**Improved knowledge (58)**						**7.7**		**.02**	
	Totally disagree/partially disagree		35 (18.9)		11 (10.2)				
	Neither agree nor disagree		37 (20.0)		35 (32.4)				
	Totally agree/partially agree		113 (61.1)		62 (57.4)				
**Changed attitude (58)**						**2.3**		**.31**	
	Totally disagree/partially disagree		38 (20.5)		16 (14.8)				
	Neither agree nor disagree		52 (28.1)		38 (35.2)				
	Totally agree/partially agree		95 (51.4)		54 (50.0)				
**Changed risk perception (58)**						**7.6**		**.02**	
	Totally disagree/partially disagree		34 (18.4)		15 (13.9)				
	Neither agree nor disagree		41 (22.2)		40 (37.0)				
	Totally agree/partially agree		110 (59.5)		53 (49.1)				
**Improved skills to reduce binge drinking (58)**						**3.8**		**.15**	
	Totally disagree/partially disagree		30 (16.2)		15 (13.9)				
	Neither agree nor disagree		47 (25.4)		39 (36.1)				
	Totally agree/partially agree		108 (58.4)		54 (50.0)				
**Would use the program again (58)**						**2.9**		**.23**	
	Totally disagree/partially disagree		34 (18.4)		15 (13.9)				
	Neither agree nor disagree		50 (27.0)		39 (36.1)				
	Totally agree/partially agree		101 (54.6)		54 (50.0)				
**Would recommend the program to someone else (58)**						**4.2**		**.12**	
	Totally disagree/partially disagree		27 (14.6)		10 (9.3)				
	Neither agree nor disagree		39 (21.1)		33 (30.6)				
	Totally agree/partially agree		119 (64.3)		65 (60.2)				

## Discussion

### Principal Findings

In this paper, the effectiveness of the first Web-based, CT intervention to reduce BD in Spanish adolescents was tested through a cluster randomized controlled trial. An overall effect of the intervention on BD was not found at 4 months, but there was an effect on reducing HED. In addition, no effects were found on weekly consumption or on any consumption.

In a similar previous study, Jander et al [16] found a significant link between condition and age, where their intervention turned out to be effective in reducing BD in adolescents between 15 and 16 years or age. In our study, although there was a trend in BD reduction in both conditions, effects of the intervention on BD behavior was not found. Nevertheless, the intervention was effective in reducing HED, in contrast to Jander et al [16], where this pattern of alcohol consumption showed a lower prevalence than in previous studies [16,54]. One explanation for our results may be that alcohol consumption is accepted in the Spanish context, including BD, whereas the more extreme type of drinking is less accepted and, thus, easier to change. As alcohol use is quite normative among Spanish adolescents [6,8,55], mostly in the context of parties or celebrations, it could be that the adolescents had no incentive to change their behaviors at these events [16,56]. In fact, in a previous qualitative study, many adolescents reported that they usually drink when they are at parties or during special occasions, such as fairs, Holy Week, or Christmas, showing a low-risk perception of BD (paper not yet published).

Moreover, in Spain, the alcohol drinking phenomenon called *Botellón* is one of the main sources of entertainment among young people, especially during nonschool periods, and it is normal for youths to binge drink at this kind of event [55,57]. Because of this, our study may have reduced the number of glasses of alcohol in the EC to be effective on HED, but it was not reduced enough to be effective on BD. Hence, future research needs to look at how to change norms regarding BD and how to prevent BD using more comprehensive campaigns and addressing cultural norms on BD. In addition, due to a lack of consensus as to what is considered an SDU [6,9,10], there may be varied results between countries, so these results may not be applicable to other populations.

Another possible explanation is that the follow-up in the EC, with respect to the CC, was closer to holiday periods, such as Holy Week or fairs, when adolescents usually go out to drink. Thus, this might be another reason related to the lack of effect on BD. Moreover, the intervention was only assessed in the fourth month; it would be advisable to carry out long-term assessments (ie, 12 or 24 months or an even longer time period) because several authors state that it requires a longer time period to see a real behavioral change [16,24].

Previous studies on CT interventions found high attrition rates [16,34,36], often caused by a lack of face-to-face contact and a high degree of anonymity [37,58]. A 50% attrition rate was taken into account in our power calculation, as with another study with a similar purpose to ours [39], and our study reported a lower attrition rate than that in other studies, such as Jander et al [16], Elfeddali et al [34], and Stanczyk et al [59], whose dropout rates were 68.9%, 62.9%, and 52.60%, respectively. Hence, strategies such as carrying out most of the sessions within the schools as part of the health promotion curriculum, collaborating with teachers at the pre- and posttest sessions by phone call, or, if necessary, assisting teachers at the schools, could be useful to minimize the dropout rate. That is why we believe that future studies should continue to carry out the intervention within the school curriculum to reduce dropout rates. Furthermore, older adolescents and those who were enrolled in higher educational-level programs dropped out more often. Moreover, those who dropped out were more likely to engage in BD and reported a higher weekly consumption. These characteristics are consistent with other studies [16,27,34,60].

The recommendations by Jander et al [16] on how to approach the intervention were taken into account in the Alerta Alcohol program in trying to improve the adherence to the intervention, which seems to show slightly better results in our study. We focused on premotivational determinants, such as knowledge and risk perception [44,61], since, in a previous qualitative study, we found that the majority of adolescents were in the precontemplation phase—they had no intention of changing their health behavior (paper not yet published). According to the Transtheoretical Model, an increase of consciousness is an important first step leading toward behavior change [62]. We also developed a dynamic intervention, with different stories adapted to gender and age [23,39]. Moreover, we followed up on the intervention at the schools (eg, by phone call or, if necessary, in-person visit). It is possible that these actions could slightly improve the adherence to the Alerta Alcohol program.

Although two booster sessions were introduced at home, there was little participation; a possible solution to increase participation may be to incorporate these booster sessions within the school. Most of the participants stated that the reminder emails arrived in their spam folders, making the emails ineffective, which explains the low participation in these sessions. Some authors highlight the importance of the content and the schedule of the reminders [63,64], as well as sending notifications by other means, such as by text message and WhatsApp, among others, so these aspects could be improved in future studies.

To improve adherence in future interventions, it is necessary to be familiar with the predictors of adherence. In our study, adolescents belonging to VT and CSE groups showed less adherence. In this sense, it should be emphasized that the adolescents who belonged to the VT group could have had a higher dropout rate because they finished the academic year before the posttest, and at the time of the posttest they were out of school. This should be taken into account for upcoming interventions. Furthermore, we found that being Spanish also appears to be a predictor of adherence. Also, the analyses of adherence indicated that non-binge drinkers adhered better to the intervention, this last finding being typical in health promotion. This is because, as a rule, people who adhere better to a health program tend to have better lifestyle habits [65]. However, other studies, such as that conducted by Schneider et al [66], found that people with an unhealthy lifestyle usually visit their health intervention website more frequently; although, it is true that people with a healthier lifestyle were more likely to complete the health intervention. The problem is that health promotion programs should focus, particularly, on improving the health and lifestyle of people who lack a healthy lifestyle, even though the people who already lead a healthy lifestyle will strengthen their own. That is why we believe that more research is needed to know how to improve the adherence of people engaging in BD.

Finally, in the process evaluation, we found that adolescents in the EC group, in general, were very satisfied with the program. Regarding BD, binge drinkers were more satisfied with the program and showed a better response. Regarding gender, we can verify that females reported better a response to the program, which could be positive, since girls reported a higher rate of BD, and the intention of the health program is to focus on those who have an unhealthy lifestyle [66]. Moreover, it could be that boys are more reluctant to change their behavior or participate in programs to improve their health.

### Strength and Limitations

The main strength of this study is that it was based on the I-Change Model for predicting healthy behavior acquisition and was preceded by quantitative [46] and qualitative research [39]. Furthermore, the Alerta Alcohol program was an adaptation of a previous intervention developed in the Netherlands based on extensive research that showed cost-effectiveness and effectiveness on BD among 15-16-year-olds [16,25,38]. Although several interventions exist regarding alcohol, few address BD, and even fewer interventions use CT technology in this target group at the school level. In addition, this study builds on earlier work done in the Netherlands, which was adapted to the Spanish context. As both replication and implementation are important for science, we are convinced that this intervention is highly relevant and innovative. Moreover, for the Spanish context, there is no similar study targeting alcohol consumption and BD prevention using a Web-based, CT intervention in adolescents. Moreover, our sample was randomized into EC and CC groups, and there were hardly any differences at baseline between these groups.

One of the limitations of this study was that only short-term outcomes of the intervention were assessed. It would be advisable to add more long-term assessments to evaluate the effects in the long run. In addition, the follow-up evaluation coincided with periods after holidays, which may have increased the probability of BD, as well as with periods in which adolescents belonging to the VT group were out of school, so this population was underrepresented. Further, many adolescents reported that some advice messages were long, therefore, they may have found them tiresome to read. In addition, it should be noted that Jander et al [16] developed their intervention in the context of a serious tailored game, which was not possible in our case, even though our program was a tailored dynamic intervention. Instead of a serious game, we used stories based on real facts identified in the previous focus group study.

Another limitation is that the sample size was determined for logistic regression, not taking clustering due to schools and classes into account. However, although we allowed for clustering in the analyses, for all binary outcomes the variances of the random effects turned out to be zero, which actually reduced the analysis to a logistic regression. Furthermore, it must be noted that we were rather close to complying with the required number of people—612 versus 618.

Moreover, compliance with the intervention protocol was relatively high for the initial classroom sessions, but this tailed off toward the end of the experimental period. However, this study improved the attrition rate through an adaptation of recommendations from the previous intervention developed in the Netherlands, resulting in a lower attrition rate—50.9% vs 68.9% [16]; high attrition rates are common in Web-based, CT interventions [67].

The large volume of missing data (>50%) was mainly due to missing values on the outcome variables, with less than 4% being due to missing values on covariates. In the case of missing values on outcomes, it is known that complete case analysis and multiple imputation yield the same results [52]. Since the fraction of missing cases due to covariates is very small, the added value of multiple imputation of these covariates is considered to be minor. It is also worth noting that, although multiple imputation might be of added value, there is also the risk of choosing an incorrect imputation model, which could lead to biased results [53].

Finally, one should be cautious when interpreting these results on the effectiveness of the program for the variable *HED*, since only 16 subjects were classified as heavy drinkers at the pretest stage.

### Conclusions

We observed that the rate of BD decreased more in the EC group than in the CC group, and the overall effect on BD was not significant. However, we did find that there was an effect on HED. We believe that using the CT intervention at schools could be a good option for reducing some alcohol patterns such as HED among adolescents. However, we must be cautious when interpreting the results due to the low number of subjects in the HED group, which could affect the generalization of the study. Further research with long-term measurements is needed, as well as improvements in adherence to eHealth interventions, which will increase effectiveness and public health impact. In this sense, as one of the strategies for improving the program was to shorten the messages, perhaps future programs could benefit from the use of more pictures and avatars. Another option is to compare written messages with video or audio messages, so that the adherence to, and effectiveness of, the program can be improved.

## Appendix

Multimedia Appendix 1 CONSORT-EHEALTH checklist (V 1.6.1).

Multimedia Appendix 2 Measurement variables from the Alerta Alcohol program.

## Abbreviations

BD: binge drinking
CC: control condition
CONSORT: Consolidated Standards of Reporting Trials
CSE: compulsory secondary education
CT: computer tailored
EC: experimental condition
ESPAD: European School Survey Project on Alcohol and Other Drugs
ESTUDES: Encuesta Sobre Uso de Drogas en Estudiantes de Enseñanzas Secundarias
FAS: Family Affluence Scale
GDPR: General Data Protection Regulation
HED: heavy episodic drinking
OR: odds ratio
SDU: standard drink unit
VT: vocational training
