# Supplementary material for: A Web-Based, Computer-Tailored Intervention to Reduce Alcohol Consumption and Binge Drinking Among Spanish Adolescents: Cluster Randomized Controlled Trial
Source: J Med Internet Res. 2020 Jan 24;22(1):e15438. doi: 10.2196/15438 (PMC7007597; doi:10.2196/15438)
Supplement: Multimedia Appendix 2 [file jmir_v22i1e15438_app2.docx]

**Appendix 2:** *Alerta Alcohol*: Measurement variables.

| **Questions** | **Answers** | **Variable type** |
| --- | --- | --- |
| **Sociodemographic** | | |
| Gender | 1 = Male; 2 = Female | Nominal binary |
| Age | It was extracted from the period between the pre-test date and the date of birth of the participant | Quantitative |
| Course | 1 = 4th ^a^CSE; 2 = 1st Baccalaureate; 3 = ^b^VT | Ordinal |
| Religion: What is your religion? | 1 = Catholics; 2 = Protestant/Evangelical; 3 = Muslim/Islam; 4= Other; 5 = No religion; | Nominal |
| Nationality: What is your nationality? | 1 = Spanish; 2 = Other | Nominal binary |
| ^c^Family Affluence Scale | It was a sum-up of the following questions: Does your family own a car or van? (1 = No; 2 = Yes, one; 3 = Two or more); How many times did you go on vacation with your family during the last 12 months? (1 = Never; 2 = Once; 3 = Twice; 4 = More than twice); How many computers does your family have? (1 = Nothing; 2 = One; 3 = Two; 4 = More than two); Do you have your own bedroom? (1 = No; 2 = Yes) | Scale |
| ^d^**Family Apgar Test:** Satisfaction level with the help that he/she receives from his/her family when he/she has any problem. | 1 = Almost never  2 = Sometimes  3 = Almost always | Nominal |
| **Family Apgar Test:** Frequency with which they talk between them about the problems that they have at home. | 1 = Almost never  2 = Sometimes  3 = Almost always | Nominal |
| **Family Apgar Test:** Frequency with which the important decisions are taken together at home. | 1 = Almost never  2 = Sometimes  3 = Almost always | Nominal |
| **Family Apgar Test:** Satisfaction level with the time that he/she is together with his/her family. | 1 = Almost never  2 = Sometimes  3 = Almost always | Nominal |
| **Family Apgar Test:** Feelings about whether his/her family loves you. | 1 = Almost never  2 = Sometimes  3 = Almost always | Nominal |
| **Alcohol use** | | |
| Days of last week he/she drank alcohol. | 1 = Monday; 2 = Tuesday; 3 = Wednesday; 4 = Thursday; 5 = Friday; 6 = Saturday; 7 = Sunday; 8 = I didn't drink alcohol last week; 9 = I never drink alcohol | Nominal |
| ^e^How many standard glasses of alcohol did you drink last week? | Monday; Tuesday; Wednesday; Thursday; Friday; Saturday; Sunday | Quantitative |
| Number of times he/she drank 4/5 standard glasses or more of alcohol in one single occasion in the last 30 days. | In the last 30 days, how many times have you consumed 4 or more standard glasses (if you are a girl) or 5 standard glasses or more (if you are a boy) of alcohol on one occasion (for example at a party or at night)? Answer with a number (eg 0, 1, 2 etc.) | Quantitative |
| ^a^CSE: Compulsory Secondary Education.  ^b^VT: Vocational Training.  ^c^Family Affluence Scale: Currie, Molcho, Boyce, et al. (2008).  ^d^Family Apgar Test: Smilkstein, Ashworth, Montano (1982).  ^e^The “weekly consumption” and “heavy episode drinking” variables were created from this variable | | |
